# Supplementary material for: Let’s Play the fMRI—Advantages of Gamified Paradigm in Examining the Motor Cortex of Young Children
Source: J Clin Med. 2022 May 22;11(10):2929. doi: 10.3390/jcm11102929 (PMC9145096; doi:10.3390/jcm11102929)
Supplement: Supplementary file 1 [file jcm-11-02929-s001.zip › jcm-1613045-supplementary Table S1.pdf]

Supplementary Table S1. Differences in the number of completed active blocks and squeezing effectiveness between the two types of paradigm.

Age (Years)	Sex	Completed Blocks (Number)		Percentage of Power Bar Uploading in Each Block									
				1		2		3		4		5	
		S	G	S	G	S	G	S	G	S	G	S	G
4	F	1 (1.2)	1.9 (0.7)	45.4 (16.9)	60.0 (12.8)	33.1 (16.7)	45.1 (17.5)	19.4 (19.3)	27.6 (16.2)	14.2 (11.8)	17.5 (11.8)	4.9 (5.5)	9.2 (9.5)
	M	0.8 (1.0)	2.0 (0.8)	37.8 (19.5)	65.2 (10.2)	39.6 (14.6)	44.8 (17.5)	27.8 (14.1)	24.0 (20.0)	23.2 (14.5)	19.3 (18.7)	10.6 (8.2)	15.9 (8.5)
5	F	2.3 (1.5)	3.2 (1.0)	56.0 (16.2)	76.1 (5.4)	52.7 (22.3)	69.0 (7.3)	45.9 (17.5)	59.4 (14.4)	33.2 (15.0)	40.3 (14.9)	19.3 (13.8)	28.0 (17.4)
	M	1.7 (1.4)	3.5 (1.3)	48.0 (16.1)	78.0 (8.5)	45.7 (13.6)	69.9 (9.7)	39.4 (18.3)	59.1 (15.8)	23.8 (14.6)	52.7 (21.0)	17.3 (14.9)	49.2 (21.0)
6	F	3.6 (1.2)	4.4 (0.5)	67.4 (7.9)	81.0 (8.0)	65.8 (5.3)	73.6 (5.3)	51.5 (12.4)	68.2 (6.8)	40.8 (14.5)	63.4 (7.5)	28.4 (18.2)	52.6 (21.0)
	M	3.6 (1.0)	4.7 (0.5)	71.3 (12.0)	83.4 (9.4)	64.4 (10.2)	75.3 (6.8)	56.9 (16.6)	70.1 (9.5)	49.2 (15.1)	67.4 (7.8)	29.2 (17.2)	59.0 (17.9)

F—female, M—male, S—standard, G—gamified.
